# Foot health among the Roma population of southern Spain according to the foot health status questionnaire

**DOI:** 10.1186/s12889-020-08571-2

**Published:** 2020-04-06

**Authors:** Manuel Coheña-Jiménez, Esther Chicharro-Luna, José Algaba-Del-Castillo, Amanda Páez-Tudela

**Affiliations:** 1 Department of Podiatry, Faculty of Nursing, Physiotherapy and Podiatry, University of Sevilla, Avicena st, 41009 Sevilla, Spain; 2 Department of Behavioral Sciences and Health, Faculty of Medicine, University Miguel Hernández, San Juan de Alicante, Spain

**Keywords:** Foot health, Roma population, Spain, Minority health, Podiatry

## Abstract

**Background:**

Foot health of the Roma population is a challenge for the health professionals where this minority is significant, as is the case in Spain. At present, little is known about foot health of the Roma population and their knowledge would promote the training of these professionals at the community level. Foot pain is common and a reason for consulting podiatry services. The purpose of this study was to determine foot health among the Roma population according to the Foot Health Status Questionnaire.

**Method:**

An observational, cross-sectional and quantitative study conducted at the Roma population living in Spain in 2018. Self-reported data and the Foot Health Status Questionnaire were recorded. Examining the general health and foot health (foot pain, foot function, footwear and general health) and general (general health, social capacity, physical activity and vigour). This questionnaire is recommended as a valid and reliable patient-reported outcome. The obtained scores were compared.

**Results:**

A sample made up of 624 men and women from the Roma population took part in this study. 45% were Roma men and 55% Roma women. In the first section of the FHSQ, a lower score of values was recorded in the footwear domain (62.5) and in the general foot health domain (60). Gypsy women obtained lower scores in all the domains. In the second section, lower scores were obtained in the vigour (56) domain and in the general health (60) domain. A large effect size (r-Rosenthal) was found by gender in the footwear domain (0.334) and in the vigour domain (0.195). Roma women showed higher values in cardiac disorders, serious illnesses, doctor visits and foot problems. 67.8% reported that they had never been assisted by a podiatrist.

**Conclusions:**

The studied Roma population has foot health problems, and these are more pronounced among women. They show lower values in the footwear and vigour domains. More professional training is required for health workers in this field to avoid cultural diversity stereotypes.

## Background

The Roma population is the oldest and largest ethnic minority in Spain [1] with their own cultural and moral values [2], and in Europe it represents one of the oldest ethnic groups (1.35% of Europe’s total population) [3]. Currently, in Europe the Roma population is estimated to be of seven to 9 million people and, in Spain, it represents 1.5–2.1% of the total Spanish population (725,000–750,000 Roma people) [4] [5]. Specifically, in Andalusia (in southern Spain) there is almost half of the population of this ethnicity. The Roma population is the ethnic group that suffers the highest discrimination rate, both in Europe and in Spain, even though data indicate that the Roma population feels that they were successfully integrated into the Spanish society, which is a plural society [3] [4]. Condon et al. reported that the Roma population has serious health inequalities when compared to other minority ethnic groups [6], and that they have poor health indicators with respect to the non-Roma population [7]. Health inequalities are due to the fact, that it is an ethnic group with its own culture and values [2], and not only that, minority groups have a socio-economic gap [8]. However, some authors point out that the absence of evidence on the health of the Roma population should not be equated with evidence on the absence of health inequalities [9].

According to this background, both in Spain and in Europe (Romania, United Kingdom, France, Belgium, Slovakia), among the Roma community, Roma women are the main ones affected by gender inequalities [4]. According to George et al., vulnerable minority populations across Europe face various barriers to accessing medical care [10]; although La-parra-Casado et al. point out that there is a real health gap, the difference in perceived health cannot be displayed as a gap. While Roma people report very poor health at a high percentage, they really show a similar proportion of very good health [1].

In 2015, Martín-Pérez et al. reported a study on the predictors on using medication in the Roma population in Spain. This study included a questionnaire with questions about health status, lifestyle and access to health services, as well as questions designed to collect demographic information about the household and its members [11]. The authors reported the presence of chronic diseases, a negative perception on health and medical consultations were associated with an increased use of medication in the study population [11].

The Roma population is a difficult group to reach in research and there is lack of visibility of their health research studies [1]. In fact, the literature is very heterogeneous and the majority deals with studies of communicable diseases and, to a lesser extent, noncommunicable diseases, and those related to health prevention are scarce. Therefore, studies on the health situation of the Roma people are necessary and timely. This was reported by Hajioff and McKee in a review of the published literature pointing out the need to improve health interventions but taking into account the social and cultural aspects of the Roma community [9].

Despite general acknowledgment of this situation, to our knowledge no previous studies have been conducted to determine the state of foot health among ethnic minorities in Spain. The promotion of foot health can enhance public health among the population in general and among the Roma people in particular. Previous research has shown that foot-related health problems, usually pain and deformities, are among the main reasons for consulting with specialists and for seeking high-quality podiatric care [12] [13] [14]. Foot health specialists are essential to implement prevention strategies in foot health and can approach the concept of prevention to the Roma population [1].

Public health is determined by various factors and is often subjected to avoidable social inequalities, often related to gender, ethnicity, etc. [15] [16]. The Foot Health Status can be defined as the state of physical, social and psychological well-being specifically related to the foot, and requires attention on the part of public health. One way to measure this concept is through a validated Foot Health Status Questionnaire, which inquires into the impact of foot status on the patient’s quality of life [17] [18]. López-López et al. reported that foot problems and quality of life in vulnerable populations have an impact on work and social life [19].

Studies have been conducted to investigate foot health in different population groups [20] [21] [22] (pregnant women, women, adolescents, diabetics, hallux abducto valgus, fibromyalgia, etc. …) but they have not been carried out according to the ethnic origin of the population. It is known that the general population has problems with deformity and pain in the feet that require visits and treatment by podiatrists, with high prevalence rates (61.3–79%) reported in institutional and clinical settings [23]. However, there is no available evidence on foot health in the Roma population. Foot conditions and deformities should be taken into account in this population, in an attempt to provide better health care throughout society. Our article is aligned with the Framework for National Roma Integration Strategies up to 2020 (European Commission, 2011), according to which Roma integration entails addressing the health care realm.

Measuring the quality of life of a population is the initial step in the approach to prevention strategies and action protocols to reduce health problems [24]. According to these authors, to date there have been no studies on the foot health needs of the minority ethnic groups. This study aimed to determine foot health among the Roma population according to the Foot Health Status Questionnaire and, consequently, we contribute to published research on the health needs of the Roma population.

## Methods

### Study population and data collection

We conducted a cross-sectional study among Roma population. This descriptive and cross-sectional study was performed with a representative sample of the Roma population in Andalusia (southern Spain), composed of 624 people self-identified as members of this community. The field work was carried out from January to December 2018.

### Study desing

It is known that research on the health of the Roma population is limited and difficult to access [6]. Therefore, to increase the validity of the study, self-administered questionnaires were completed with the help of the Spanish Roma foundation. The sample was selected randomly among the individuals who attended the different provincial headquarters of the Spanish Roma foundation. The sample randomization carried out flipping a coin. This organization has previously participated with the Ministry of Health and Consumer Affairs of Spain and carried out the first National Health Survey among the Roma population in Spain [25].

Almost all the interviewers worked directly or indirectly with the Roma population. La-parra et al. reported that many of barriers (language and age barriers, lack of citizenship, lack of recognition of the right to health) do not exist in the Spanish case because the Spanish Roma are Spanish citizens, they speak the language of the majority [1]. The interviewers helped people with doubts about a question and/or those who had vision problems because they did not have glasses at that time, and had difficulty reading the document. In addition, they favoured a climate of trust and interpersonal communication. The tool used is not adapted to the Roma population, since it is known that the Roma culture has existed without a written language and that they have transmitted their culture orally from one generation to the next, with its written adaptation not being necessary [25]. The use of this technique proved to be a good decision, since most Roma population were able to complete the questionnaires.

### Inclusion and exclusion criteria

The basic criteria for inclusion in the study sample were age of at least 18 years old and the ability to read, understand and complete the questionnaire. Participants under the age of 18 and/or those with cognitive problems were excluded. Some participants did not complete the questionnaire. We declared that they were unable to complete the questionnaire, they were excluded from the study. Likewise, those people who voluntarily did not want to participate did not do so. It takes the respondent less than 10 min to complete.

### Sample size determination

The formula for the descriptive estimation of a single proportion has been used to obtain the sample size. In order not to assume any specific value, the proportion is set at 0.5 (*p* = 0.5) (q = 1-p = 0.5). The proportion was estimated and set at 6% (FE = ±0.06). For a 95% confidence interval, the error was set at α = 2.5% in each queue (α/2 = 0.025) and a Z value of 1.96. And after applying the following equation:

$$ n=\frac{z_{\alpha /2}^2\bullet p\bullet q}{FE^2} $$= $$ \frac{1,{96}^2\bullet 0,5\bullet 0,5}{0,{06}^2}=\mathrm{266,78}\sim 267. $$

It is concluded that a sample size of 267 subjects will be needed, for each group to be analysed. In our study, when making comparisons between men and women, at least 267 Roma men and 267 Roma women are needed. In this study, 624 participants were recruited in consideration of possible losses.

### Research desing

We use the measurement of the questionnaire survey: FHSQ. The study data were obtained by means of the short-form Foot Health Status Questionnaire (version FHSQ 1.03), which is self-administered and has been validated in previous research, on the quality of life related to foot health [17] [18] [21] [26] [27]. This questionnaire, developed and validated in Australia, is increasingly used in foot and ankle research [28] [29]. The Spanish version of the FHSQ showed adequate psychometric properties [26].

The FHSQ contains three sections and evaluates the following domains: “foot pain” (type, severity and duration: four items); “functional capacity of the foot” (four items); “footwear” (lifestyle and difficulty in the use of footwear: three items); and “general foot health” (self-perceptions and foot condition: two items), overall health, physical function, social capacity and vigour [18]. The first section evaluates foot health with 13 questions reflecting four domains relating to foot health: foot pain, functional capacity, footwear, and general foot health. The evaluation of footwear uses practical aspects of shoe comfort. Each question allows several answers, and these are placed on a Likert-type ordinal scale and the descriptors for these scales vary for each domain (appendix 1). The second section evaluates four domains related to general health: overall health, physical activity, social capacity and vigour (appendix 2). The third section contains questions that request sociodemographic data (gender, age) and questions about their medical records and the use of the health service, toxic habits and satisfaction information [17] [18] [27]. The perception of general foot health is based on the patients’ self-assessment of the state of their feet, as a subjective variable of self-reported health [8]. The questionnaire does not provide an overall score. To obtain these indexes, the responses are analysed through computer software (FHSQ, version 1.03). After processing the data, the software produces a score ranging from 0 to 100. The maximum score is indicative of a person with excellent foot health, no pain or discomfort and no difficulty or limitations in putting on shoes, performing activities of daily life or work, with adequate functional capacity; a score of zero represents the worst state of health for the foot [17]. The first section has demonstrated high degrees of content, criterion and construct validity (Cronbach’s α = 0.89–0.95) and high retest reliability (intraclass correlation coefficient = 0.74–0.92). The questions in the second section are adapted from the Medical Outcomes Study 36-Item Short-Form Health Survey, which has been shown to be valid [27] [30]. The final section of the questionnaire has items on general health status, with yes/no response options, related to cardiac alterations, diabetes, communicable diseases, arterial hypertension, serious illnesses, allergies, recent surgical interventions, consumption of medication and use of alcohol and/or tobacco.

In addition, the study analysed the opinion of the participants in the questionnaire and, to assess the use of health care resources related to foot care, the participants were asked about visits to a podiatrist in the last 3 months, and their preference for public or private podiatry services. The final questionnaire items asked about the respondent’s degree of satisfaction with the questionnaire as regards the difficulty in answering, the comprehensibility of the questions, the content and language used, and the treatment received during the interviews.

### Statistical analysis

The statistical analysis was performed using the IBM SPSS Statistics v. Twenty two statistical software. The descriptive data provided absolute frequency values and percentages for categorical variables, and median and interquartile range depending on the variables. The Chi-square test has been used to see if there is any kind of relationship between the qualitative variables. The Mann–Whitney’s U test was used for the independent samples when there was no normal distribution. An inferential analysis was carried out taking into account and a priori level of confidence of 95%, so the experimental *p*-value has been compared with a significance level of 5% and the tests applied have been non-parametric. When statistically significant differences were found according to the p-value, the effect size was calculated using Rosenthal’s r to analyse the magnitude of the differences [31]. The Foot Health Status Questionnaire version 1.03 was used to obtain scores relating to foot health. We followed the Strengthening the Reporting of Observational Studies in Epidemiology (STROBE) guidelines for the presentation and writing of our manuscript.

## Results

The study sample was composed of 624 men and women from the Roma population resident in Andalusia (Spain). 45% (*n* = 278) were men and 55% (*n* = 346) women, and their mean age was 40.96 years old (SD: 17.58). Forty eight participants did not complete the questionnaire.

Table 1 Comparison of the FHSQ scores of the total sample and between Roma men and Roma women. The first section evaluated four specific foot domains (pain, function, health, and footwear). The median scores were higher in relation to the assessment of pain and function, and lower in relation to foot health and footwear. The median foot pain scores for men and women were 78.75 and 72.5, respectively. The foot function score was 81.25 for men and 68.75 for women. The overall health of the foot score was 61.25 and 60 for men and women respectively. The lowest value of this section was for the score of the footwear women with a value of 25, the men obtained a value of 75 (Table 1).

**Table 1:** Comparison FHSQ scores of the total sample and between Roma men and Roma women

FHSQ domains	Total group ***n*** = 624 *Median ± IR Range (min-max)	Roma men n = 278 *Median ± IR Range (min-max)	Roma women ***n*** = 346 *Median ± IR Range (min-max)	p-valor	r-Rosenthal Effect size
Foot Pain	75 ± 26.88 (0–100)	78.75 ± 27.5 (12.5–100)	72.5 ± 33.28 (0–100)	*< 0.001*	0.175
Foot Function	75 ± 37.5 (0–100)	81.25 ± 37.5 (0–100)	68.75 ± 32.81 (0–100)	*< 0.001*	0.175
Footwear	62.5 ± 50 (0–100)	75 ± 50 (0–100)	25 ± 50 (0–100)	*< 0.001*	0.334
General Foot	60 ± 42.5 (0–100)	61.25 ± 30 (0–100)	60 ± 55 (0–100)	*< 0.001*	0.175
Health General Health	70 ± 40 (0–100)	70 ± 40 (0–100)	60 ± 40 (0–100)	*0.001*	0.137
Physical activity	94.44 ± 22.22 (0–100)	97.22 ± 16.67 (0–100)	94.44 ± 27.78 (0–100)	*< 0.001*	0.142
Social capacity	87.5 ± 37.5 (0–100)	87.5 ± 25 (0–100)	87.5 ± 37.5 (0–100)	*0.006*	0.110
Vigour	62.5 ± 25 (0–100)	62.5 ± 25 (0–100)	56.25 ± 25 (0–100)	*< 0.001*	0.195

*Median ± IR = Median ± interquartile range

The second section evaluated four domains of general well-being: overall health, physical function, social capacity, and vigour. The highest scores were for the physical activity domain of men with 97.22, and 94.44 for women. The social capacity domain obtained the same score (87.5) both men and women. Women’s scores for the foot health in general domain and for vigour were lower than those for men. Women showed values of 60 and 56.25, and men of 70 and 62.5, for overall health and for vigour respectively. Roma women showed significantly lower scores in the various domains (*p* < 0.001), and the social capacity domain was *p* = 0.006. A large effect size by gender was found in the shoe domain (0.334). The size of the effect was very small for social capacity, with a value of 0.110. (Table 1).

Table 2 Clinical characteristics for the Roma population sample. The highest values were for doctor visits (71.5%), vaccines (69.6%) and foot problems with 68.8% (429 participants). These foot problems are found in 249 women (72%) and 180 men (64.7%), respectively. Almost 50% report taking medication (42.4% men and 48.8% women). The lowest values are for the question about whether they have any communicable disease (0.3%). Almost 21.7% of the participating Roma women and 41.4% of the Roma men reported toxic habits (*p* < 0.001), such as alcohol or tobacco consumption. In contrast, Roma men reported more positive results and consider their foot health to be very good.

**Table 2:** Clinical characteristics of the sample Roma population

	Total n = 624		Roma-men n = 278		Roma-women n = 346		p-valor
	n	%	n	%	n	%	
Cardiac alterations*	52	8.3	17	6.1	35	10.1	*0.048*
Diabetes	72	11.5	34	12.2	38	11.0	0.359
Communicable disease	2	0.3	1	0.4	1	0.3	0.693
Arterial hypertension	123	19.7	59	21.2	64	18.5	0.227
Serious illness*	63	10.1	20	7.2	43	12.4	*0.021*
Allergy	154	24.7	66	23.7	88	25.4	0.347
Vaccine	434	69.6	198	71.2	236	68.2	0.234
Surgery	104	16.7	39	14.0	65	18.8	0.069
Medical Visit*	446	71.5	184	66.2	262	75.7	*0.006*
Medication	287	46.0	118	42.4	169	48.8	0.065
Toxics Habits*	190	30.4	115	41.4	75	21.7	*< 0.001*
Problems feet*	429	68.8	180	64.7	249	72.0	*0.033*
Visit podiatrics 3 months	87	13.9	41	14.7	46	13.3	0.342

N, absolute values; %, percentages; Chi squared test Χ^2^

Of the total sample, 67.8% reported that they had never been assisted by a podiatrist. 92.3% were in favour of receiving a free service. Likewise, 14% of the participants acknowledged having visited a private podiatrist during the last 3 months. Almost 90% of the participants had no special difficulties to complete the questionnaire, 78% fully understood the questions and 57% considered the content of the questions interesting.

## Discussion

Because foot problems are frequently observed in the general population [22], the objective of our study was to determine foot health in the Roma population using the Foot Health Status Questionnaire (FHSQ). This is an initial study of the health needs of the Roma population considering that the Roma ethnicity is a different risk factor, independent of the sociodemographic state [32]. This is an under-researched population, and as such the work is of interest. National health surveys of the Roma population in Spain and Europe have been carried out within the framework of the European project entitled “Health and the Roma community, analysis of the situation in Europe” [9] [11] [33] but have not taken into account foot health.

There indeed have been other studies of general health [1] [8] [11] [15] [34] and more specifically of visual health [35] in the Roma population, indicating greater limitations compared to the non-Roma population. It is difficult to compare the impact of our findings with that of previous studies, as to our knowledge this is the first such analysis to be conducted of a Roma population, although similar studies, considering foot pathologies among other population groups, have been reported, highlighting the need for proper foot care in order to prevent the onset of diseases and deformities. There are numerous studies that have used this questionnaire (FHSQ) [17] [27] [28] [29] [36], which has been adapted and translated to know foot health in certain pathologies [18] [23] [24], and on populations with certain characteristics [19] [20] [22].

This research is based on people’s perceptions of health and well-being. Foot problems affect the quality of personal life, social life and the work experience [19]. Roma women show lower values than Roma men in all the FHSQ domains, with the lowest value being the footwear domain. The results of the present study confirm that Roma women present lower scores in those domains related to foot pain, social function, and vigour. Roma women experience greater challenges than men in activities of daily living and in the work environment due to foot problems, which makes walking difficult and impacts on their health in general. When analysing the domain of social capacity between Roma men and Roma women to find out the effect size, we found the lower value of 0.110 and the domain of general health with an effect size of 0.137.

This is an individual research which has displayed some new and interesting findings in respect of the impact of foot health problems on Roma population. This study has been the first to examine the impact related to foot health in a sample of the Roma population with the help of scores obtained with regard to foot health. The research should always examine the health-related outcomes and social side effects of Roma care practices. The results of this study confirm the published data about foot problems in the general population and shows that this population is subjected to foot health problems. Many women in the study population reported having difficulty in finding suitable footwear, in contrast to the men, who had no such difficulty (effect size of 0.334). In line with previous studies which have shown that health problems often affect men and women unequally, our data confirm the hypothesis that women are more prone than men to foot health problems.

The questionnaire results highlight the presence of significant differences between these Roma men and Roma women concerning the type of footwear used, which can be considered a health risk factor and a potential cause of foot pathologies [37] since foot pathologies represent a public health problem including the most vulnerable populations, as the Roma population [38]. Our results are shown in line with the study by Ramos-Morcillo and others who indicated a greater vulnerability in Roma women [39]. It highlights the existence of marked differences in foot health between Roma men and women, with the latter being more likely to suffer higher levels of pain and more frequently, like so a long-term phenomenon. Thomas et al. reported a systematic review about foot pain concurring with our results, also recognizing that the prevalence of foot pain was higher in women than in men [40].

In addition, previous studies have indicated that the comorbidity analysis was related to the poor state of the Roma people health by gender and related to unhealthy habits and lifestyles [11]. Our results report data on noncommunicable diseases, unlike many of the investigations in Roma adults who have focused more on communicable diseases [9]. In addition, the findings are consistent with previous research by other authors [41], where Roma men had a higher prevalence of chronic diseases [9] [39]. Specifically, our results indicate the presence of diabetes (12.2%) and arterial hypertension (21.2%) and toxic habits related to alcohol and tobacco consumption (41.4%) in Roma men. With regard to medication, the Spanish National Health Survey states that 69.1% take medication, while our results offer lower values of medication intake (46%), although the type of medication taken was not specified. Martín-Pérez et al. found that 37% of the Roma population had self-medicated.

Vozarova et al. compared cardiovascular diseases and diabetes in the Roma and non-Roma population reporting a high prevalence in Roma population [42], in line with Ramos-Morcillo et al. [39]. Furthermore, Palomo-López et al. reported that foot problems, including those arising from chronic diseases such as diabetes, can provoke limitations, deformities and disabilities [24]. The prevalence of foot health problems was higher in Roma women than in Roma men and such disorders can be prevented or minimized, among other measures, by adopting a healthy lifestyle “healthy behaviour” and by entrusting foot care to qualified specialists.

Our analysis shows that this population is subjected to foot health problems. It should be noted that the Roma community in Spain has free access to health care, as the Spanish National Health System offers universal coverage. At present, the clinical practice in podiatry is mainly private, and so access to foot care is largely governed by socioeconomic status. Similar conclusions have been reached regarding inequalities in the use of dental health services [43] [44] [45], in access to services related to oral health in England [46] and Sweden [47], hearing aids and visual problems [35], and in the acquisition of health care products.

These and other research findings highlight the difficulties and inequalities that may be encountered by Roma men and Roma women in accessing health care services and medical treatments [43] [48], although La-parra et al. point out that the Roma population goes to the medical services more for treatments than for preventive consultations [1]. In our opinion, these findings are important, since pain is a frequent symptom and avoiding foot services for financial reasons is the most important factor that contributes to worsening foot health.

In line with other authors, our study reveals that for many Roma people, it is only when symptoms of foot pathology become apparent that they seek treatment, and that they only tend to regard ‘good’ treatment as treatment involving practical technical procedures [1] [5]. Of the total sample, 13.9% of the participants acknowledged having visited a private podiatrist during the last 3 months. A large majority of our study population were in favour of foot care and treatment being provided free of charge within the public health system. That is in line with that is set out by other authors like La-parra et al. regarding that the disadvantaged socioeconomic groups receive less health coverage and consequently are more exposed to foot health problems [49].

Studies such as ours contribute to improving health care strategies aimed at the Roma population, which with respect to foot health has not been considered previously [1] [50]. Our results show that Roma men and Roma women, like the population in general, wish to receive professional advice about their health problems [51]. This paper contributes valuable new information on a previously-unpublished aspect of health status in the Spanish Roma population, and may be useful for community strategies and programs for health promotion and disease prevention, both in general and with specific reference to foot care.

To our knowledge, this study is the first of its kind to investigate the question of foot health in an ethnic minority such as the Roma population. Finally, this paper provides insight into the foot problems the most vulnerable from different ethnic backgrounds within one community face when accessing healthcare.

The strength of our study is that it comprises a considerable representative sample of a hard-to-reach population, although our results should be generalized with caution, as the Roma population is a very heterogeneous group. The unwritten nature of the Roma culture, which has resulted in unwritten laws, gives rise to distrust and rejection with regard to documents, a situation which does not occur with our research. It is also necessary to consider the limitations of this study. First, we have not found studies in the literature with which to compare foot health in vulnerable Roma populations. The second limitation is that the study sample belongs exclusively in a region of southern Spain, which would indicate the need for a nationwide investigation. The third is that the conclusions of this study may be limited to those individuals who have been asked whether or not they had received a podiatry visit in the last 3 months, regardless of longer time intervals. The last limitation derives from the fact that podiatrist care is usually private, and treatment is expensive, making access to comprehensive podiatric treatment difficult for people with limited socioeconomic resources. The design of the study entails another drawback, a qualitative study would allow to know in depth the quality of the answers and to analyse the health problems of the foot. Future research in this field should take into account the types of foot pathology that are most frequent in this ethnic group, in order to improve knowledge. No further analysis of non-respondents was conducted.

## Conclusions

The studied Roma population has foot health problems, a high percentage of the Spanish Roma population has poor foot health. Roma women show lower values in the domains of footwear and vigour with respect to Roma men. With regard to the footwear used and its characteristics, Roma women indicate that they have difficulty finding comfortable shoes. In addition, almost 82% found the subject interesting, a finding that highlights the considerable interest among the Roma population about their health status. There is lack of visibility of their health research studies like this. More professional training is required for health workers in this field to avoid cultural diversity stereotypes.

## Data Availability

The datasets generated and/or analysed during the current study are not publicly available due to ethical restrictions related to participant consent obtained at the commencement of the study. An ethically compliant data set may be available from the corresponding author on reasonable request. Please contact mcohena@us.es with any data requests.

## Appendix 1. FSQH First section of the questionnaire

This section contains these questions:

1. What level of foot pain have you had during the past week?

2. How often have you had foot pain?

3. How often have you had feet ache?

4. How often did you get sharp pains in your feet?

5. Have your feet caused you to have difficulties in your work or activities?

6. Were you limited in the kind of work you could do because of your feet?

7. How much does your foot health limit you walking?

8. How much does your foot health limit you climbing stairs?

9. How would you rate your overall foot health?

10. It is hard to find shoes that do not hurt my feet.

11. I have difficulty in finding shoes that fit my feet.

12. I am limited in the number of shoes I can wear.

13. In general, what condition would you say your feet are in?

## Appendix 2. FSQH Second section of the questionnaire

This section contains these questions:

14. In general, how would you rate your health:

15. The following questions ask about activities you might do during a typical day. Does your health now limit you in these activities?

a. Vigorous activities, such as running, lifting heavy objects, or (if you wanted to) your ability to participate in strenuous sports.

b. Moderate activities, such as cleaning the house, lifting a chair, playing golf or swimming.

c. Lifting or carrying bags of shopping.

d. Climbing a steep hill.

e. Climbing one flight of stairs.

f. Getting up from a sitting position.

g. Walking more than a kilometer.

h. Walking 1 hundred meters.

i. Showering or dressing yourself.

16. This question asks to what extent your physical health or emotional problems have interfered with your normal social activities with family, friends, neighbours or social groups.

17. These questions are about how you feel and how things have been with you during the past month. For each question, please give the one answer that comes closest. How much of the time during the past 4 weeks:

a. Did you feel tired?

b. Did you have a lot of energy?

c. Did you feel worn out?

d. Did you feel full of life?

18. During the past 4 weeks, how much of the time have your emotional problems or physical health interfered with your social activities (like visiting with friends, relatives, etc.)?

19. How true or false is each of the following statements for you?

a. I seem to get sick a little easier than other people.

b. I am as healthy as anybody I know.

c. I expect my health to get worse.

d. My health is excellent.

## Abbreviations

FHSQ: Foot Health Status Questionnaire
